# Announcement: Journal of Structural Biology: X – Paper of the Year

**DOI:** 10.1016/j.yjsbx.2024.100116

**Published:** 2024-10-30

**Authors:** Natalie Reznikov

**Affiliations:** Deparment of Bioengineering, Faculty of Engineering, McGill University, Montreal, QC, Canada



The hierarchical structure of bones was seriously assessed at the end of the 17th century by the astute English physician Clopton Havers. His most influential work, *Osteologia nova,* described for the first time the microscopic structure of bone within its hierarchical context (Havers, 1691). He empirically defined bone composition as a mixture of ‘salt’ and ‘earth’ — equivalent to what we now call mineral and organics. He also described three hierarchical levels of bone organization: entire bones, cancellous and compact bone, and microscopic laminar structures in the shafts of cow bones. Since then, our understanding of both the composition and structure of bone has become much more nuanced, largely attributable to the development of microscopy techniques (Reznikov et al., 2020a). Different volumetric imaging techniques now render their own account of 3D bone structure, defined by the volume of interest and the resolution of features. Beyond 3D structural information about the bone extracellular matrix and cells, imaging is now complemented by crystallography and spectroscopy methods. Together, they probe not only elemental composition, but also immediate neighborhoods of molecular assembly structure for both organics and biomineral. Such a rich multidimensional landscape of information is as fascinating as it is overwhelming. Are denser bones stronger? Can compositional deviations be compensated for by structural features? Is crystallographic perfection of the mineral advantageous? The answers to these questions are at the same time both yes and no, depending on the context — when, where, for what? — as well as the size scale of the inquiry. Thus, a challenge for any bone researcher is to always carefully consider the vast information landscape, and to be able to navigate it nimbly.

Daniel J. Buss started his PhD as an aspiring biomedical engineer and finished it as a naturalist, a microscopist and an artist. Although bones form and grow molecule-by-molecule, from the bottom up, a 3D visualization/analysis experiment must be planned from the top down, including rich functional context to fine structural features. Dan’s special skill was to link microcomputed tomography to various electron microscopy methods (including focused ion beam scanning electron microscopy) into a streamlined cascade where the features of one structural level sequentially become the context of the next hierarchical level, across many scales (Buss et al., 2020, Buss et al., 2023a, Buss et al., 2023b). As a chess master thinks multiple moves ahead, Dan would design his multiscale 3D microscopy experiments into a strategic top-to-bottom, macroscale-to-nanoscale, pipeline allowing bidirectional re-tracing to correlate structural assembly in mineralized tissues. Besides 3D image acquisition, there are two more key pillars for successful, accurate interrogations: image processing and image interpretation. The sheer size of 3D structural data acquired in a single multiscale microscopy experiment leaves one with little choice but to ‘surrender’ to unsupervised algorithms like convolutional neural networks for 3D image segmentation (Reznikov et al., 2020b). When properly executed, these enable quantitative analyses and intuitive visualizations worth every effort. In our era of artificial intelligence, digital image processing is still an art, more than it has ever been! Finally, attentively sifting through the body of literature dating a century back to D’Arcy Thompson and Pettigrew, enabled Buss to assemble the fragments of the information landscape into one panoramic view of the hierarchical organization of bone in 3D. As Buss’s mastery of microscopy, creativity in image processing, and humility in exploring previous work all converged, he and colleagues synthesized that into the graphical structural biology review *Hierarchical organization of bone in three dimensions: A twist of twists* (Buss et al., 2022).

As the review describes, one can now name and quantify 12 hierarchical levels in the structure of bones. From the bottom up, indeed from the ‘salt’ and ‘earth’ of Havers, there are amino acids and mineral ions, both interfacing with water (Level 1). At the nanometer scale, amino acids form collagen *helices,* and calcium and phosphate ions form crystallite unit cells (Level 2). Collagen triple *helices* and tiny, imperfect, elongated crystallites form Level 3, a point where noncollagenous molecules enter the picture. Crystallites assemble laterally into polycrystalline irregular and gently *twisted* platelets (Level 4). At the scale of tens of nanometers, triple helices of collagen form *superhelical* microfibrils, whereas mineral platelets assemble into stacks: it is impossible to tell at this Level 5 where one entity ends and the other begins, as both mineral and organic structures are continuous and intertwining. At the micrometer scale, microfibrils form *rope-like* bundles that contain tessellated polycrystalline, ellipsoidal mineral assemblies (tesselles) (Level 6). Layers of mineralized orderly collagen bundles are engulfed within a thin feltwork of disordered collagen that accommodates bone cells (Level 7). A layer of bundles forms a lamella (Level 8). Lamellae *wind* into submillimeter concentric osteons, or a patchwork of lamellar packets (Level 9). Osteonal bone forms compact bone, and lamellar packets form trabeculae of cancellous bone (Level 10). Whole bones (Level 11) assemble into a skeleton (Level 12). By way of analogy, if one wishes to reproduce the same extent of nested hierarchy, say, for a human dwelling (a room in an apartment, which is in a building, which is on a street, etc.), then Level 12 would be the universe. This is a formidable hierarchy, in part deciphered by the fine work of Daniel J. Buss.
